# Neuroendocrine Tumor Metastases to the Breast: A Case Report and Review of the Literature

**DOI:** 10.7759/cureus.40703

**Published:** 2023-06-20

**Authors:** Jose A Urrego Díaz, Marcela González, Alfredo Ernesto Romero-Rojas, Jonathan Strosberg, Paola Jiménez-Vásquez

**Affiliations:** 1 Internal Medicine, Universidad Nacional de Colombia, Bogotá, COL; 2 Radiology, Instituto de Diagnóstico Médico (IDIME), Bogotá, COL; 3 Pathology, Los Cobos Medical Center, Bogotá, COL; 4 Oncology, Moffitt Cancer Center, Tampa, USA; 5 Gastrointestinal and Neuroendocrine Tumors, Centro de Tratamiento e Investigación Sobre Cáncer Luis Carlos Sarmiento Angulo (CTIC), Bogotá, COL

**Keywords:** case reports, gallium ga 68 dotatate, cecum, ileum, breast, neoplasm metastasis, neuroendocrine tumors

## Abstract

Breast metastases from neuroendocrine neoplasms (NENs) are considered infrequent. We report a case of a patient with ileocecal neuroendocrine tumor (NET) metastases to both breasts, for whom the initial clinical presentation was chronic diarrhea. Breast metastasis was initially suspected by a 68-Gallium DOTANOC positron emission tomography (PET)/CT and was confirmed by histopathology. We also performed a literature review in which we identified 116 cases of NENs metastatic to the breast reported so far. Most cases occurred in older women, were caused by NETs, and had the gastrointestinal tract as the primary site.

## Introduction

Neuroendocrine neoplasms (NENs) are tumors derived from the diffuse neuroendocrine system, which is why they can originate from most organs [1,2]. However, the most common primary sites are the lungs, small bowel, colon, and rectum. NENs is an orphan disease with an annual incidence of three to five cases per 100,000 inhabitants [3], accounting for only 0.5% of all malignancies [4,5]. They are divided into two groups: well-differentiated neuroendocrine tumors (NETs) and poorly-differentiated high-proliferation neuroendocrine carcinomas (NECs) [3,6]. In turn, NETs can be graded as low, intermediate, or high grade according to the mitotic rate and Ki-67 index [2,7], whereas NECs are always high-grade tumors [4,6]. The median survival after NEN diagnosis is 9.3 years with important variations according to geographic regions, grade, stage, histologic findings, and primary site [1,8-11].

Around 20% and 38% of patients will have metastases at diagnosis and on follow-up, respectively [1,5]. This proportion has been declining over time [12], but tends to be higher in males, non-Hispanic Whites, and in higher NEN grades [9]. The most common metastatic organs are the liver, lymph nodes, and bones [6,13]. On the other hand, breast metastases are considered infrequent; given that around 1-2% of breast malignancies are metastases [14,15] and that around 0.5-1% of metastases to the breast come from NENs [16], it could be estimated that 0.005-0.02% of breast malignancies are metastases from NENs. To our best knowledge, 116 cases have been reported on this so far, in which the report of 22 cases by Mohanty et al. is the largest series so far [17]. Nevertheless, it is likely that the real number has been underestimated both in clinical practice and in scientific reports [13,18,19]. In this report, we describe a NET originating in the ileocecal junction with metastases to both breasts. Also, we undertake a literature review with the aim of garnering insights pertaining to this population.

## Case presentation

A 50-year-old female presented with chronic diarrhea, up to four times a day, Bristol 5-6, with abdominal pain. She had a family history of Hodgkin’s lymphoma, colon, and thyroid cancer, with no other relevant history. Her physical examination was unremarkable but occult blood in stool was detected by guaiac test. She underwent a colonoscopy, which identified a giant sessile polyp on the cecum. Polyp biopsy revealed a well-differentiated NET with a 4% Ki67 index and zero mitoses per 10 high-power fields (HPF). It was positive for chromogranin and synaptophysin, while it was negative for PAX8. Staging was made with a chest and abdomen CT which showed a neoplastic thickening of the cecum walls and an ovarian mass. An octreotide scan (Octreoscan™; Mallinckrodt Pharmaceuticals, Staines-upon-Thames, United Kingdom) was also performed, which showed overexpression of somatostatin receptors at the ileocecal valve. Further workup revealed increased levels of chromogranin A (CgA) and 5-hydroxy indoleacetic acid (5-HIAA), 477 ng/ml and 96.5 mg/24h, respectively, with no other remarkable findings.

**Figure 1:**
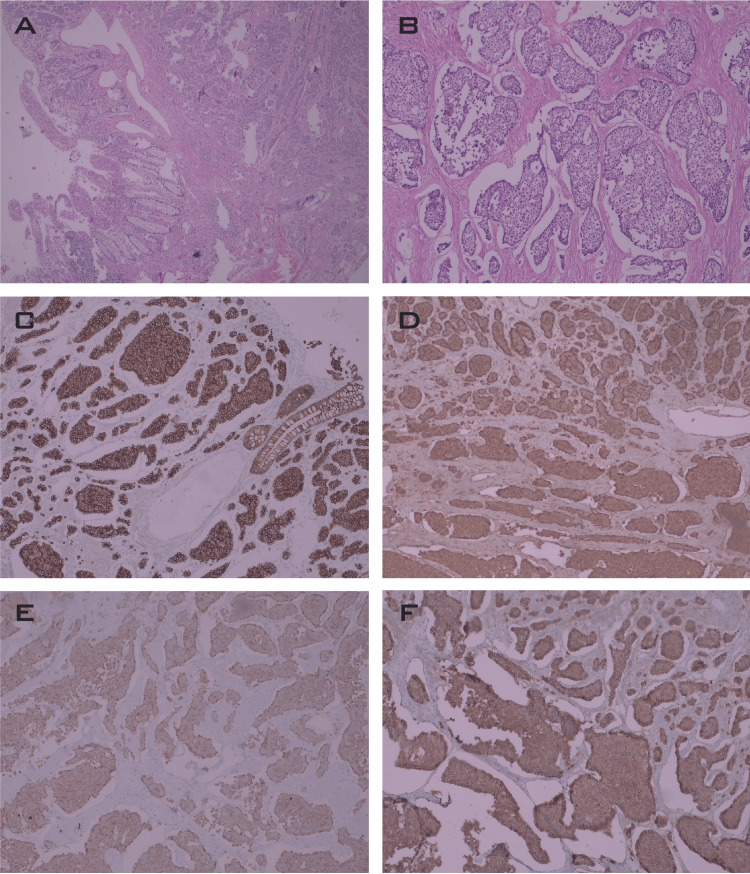
Histopathological findings of right hemicolectomy (a) H&E, 4X, intestinal wall with well-differentiated neuroendocrine tumor; (b) H&E, 40x, nests of monomorphic cells with “salt and pepper” chromatin; (c) CKAE1AE3 positivity; (d) C5, chromogranin positivity; (e) diffuse synaptophysin expression; (f) CD56 positivity.

A right hemicolectomy, bilateral salpingo-oophorectomy, and peritoneal biopsies were performed, during which invasion of serosa in the ascending colon was identified as well as malignant-appearing lesions in the small bowel, mesentery, and both ovaries. Histopathology revealed a 3 X 3 cm tumor with full-thickness invasion of the colonic wall, as well as nodules in the peritoneum, mesentery, ileum, and ovaries. It was consistent with a well-differentiated grade 2 NET, with two mitoses per 10 HPF and a 4% Ki67 index. Immunohistochemistry showed positivity for chromogranin, synaptophysin, CD56, cytokeratin AE1, AE3, and CDx2, and negativity for TTF1 (Figure 1). The patient’s diarrhea improved after surgical resection, as well as her levels of CgA and 5-HIAA, which decreased to 60 ng/ml and 6.1 mg/24 hours, respectively.

**Figure 2:**
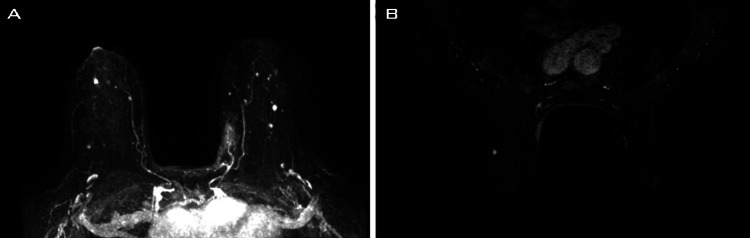
Breast contrast-enhanced MRI (a) MIP reconstruction shows bilateral multiple diffusely-distributed oval-shaped nodules with well-defined borders and marked homogeneous enhancement; (b) Subtraction imaging shows the biggest nodule at the upper inner quadrant, in which biopsy was performed. MIP: maximum intensity projection

**Figure 3:**
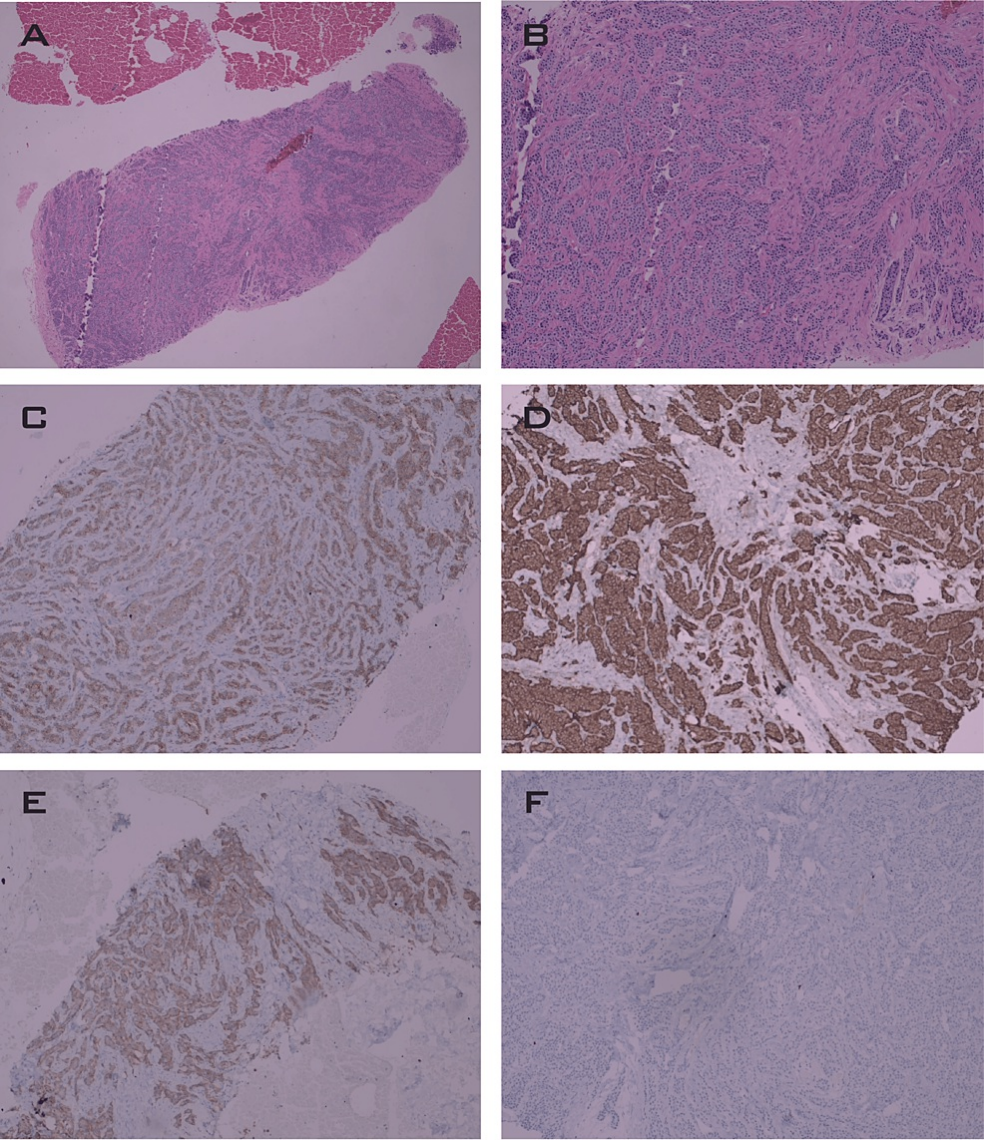
Histopathological findings of breast biopsy (a) H&E, 4X, diffuse well-differentiated NET compromise with effacement of normal breast tissue; (b) H&E, 40X, tumor with similar histologic features to those of intestinal NET; (c) CKAE1AE3 positivity; (d) Strong chromogranin positivity; (e) Diffuse synaptophysin expression; (f) Low expression of Ki67 NET: neuroendocrine tumor

A 68-gallium DOTANOC positron emission tomography/CT (68Ga-PET/CT) was performed one month after surgery and it identified somatostatin receptor expressing metastases in the liver, the recto-uterine ligament, and in a left breast nodule. A breast MRI revealed multiple, bilateral, well-circumscribed, oval-shaped nodules with homogeneous enhancement, which were classified as Breast Imaging Reporting & Data System (BI-RADS®) 4 (Figure 2). Percutaneous biopsy from the largest nodule was obtained and confirmed metastatic neuroendocrine tumor (Figure 2b, Figure 3). After the 68Ga-PET/CT results, octreotide long-acting release (LAR) 30 mg every four weeks was initiated.

For two years, the patient remained asymptomatic, except for occasional episodes of low-intensity abdominal pain. In addition, she did not develop new findings on physical examination or on routine blood chemistry. Follow-up MRI confirmed a reduction in the size and number of breast nodules, while follow-up CT did not reveal new findings on the chest, abdomen, or pelvis. However, a somatostatin receptor expressing new mass adjacent to the colon anastomosis and uterine bed was detected on a follow-up 68Ga-PET/CT. Therefore, a locoregional relapse was considered and the octreotide LAR dose was increased to 40 mg. The patient has been receiving this treatment for 18 months, without symptoms, carcinoid syndrome, and without increased levels of CgA and 5-HIAA. New images are consistent with stable disease.

## Discussion

The most common site of NETs metastases is the liver, followed by lymph nodes, bone, lungs and peritoneum [6,13,16]. On the contrary, breast metastases have been considered an unusual presentation.

We performed a comprehensive literature review in which we identified 116 reported cases of NENs metastatic to the breast, nearly all from case reports or case series (Table 1). We found that many features of our case were compatible with previous reports: (i) the mean age of the reported cases was 56 years (range 47-75 years), which was comparable to our patient's age; (ii) 89% of them were caused by NETs, while 11% were NECs; (iii) in most cases (74%), breast metastases were not the initial manifestation of the NEN (in some of the remaining cases, the tumor was initially managed as primary breast cancer); (iv) there appears to be no preferred laterality: 42% were left sided, 41% were right sided, and 16% were bilateral; (v) 40% of the cases we found developed symptoms attributable to carcinoid syndrome; (vi) nearly all cases occurred in women, with only one male case reported; (vii) in the 36 cases (31%) where metastases to other organs were reported, liver (79%), ovaries (21%), and peritoneum (12%) were the most commonly affected, just like in our case.

**Table 1:** Reported cases of NENs metastatic to breast. NEN: neuroendocrine neoplasm; NET: neuroendocrine tumor; NEC: neuroendocrine carcinoma; CR: case report; CS: case series; NaR: narrative review; CrS: Cross-sectional; NR: non-reported. ^a ^References included in the review by Upalakalin et al. were not included in the table. ^b ^Mean or median reported by authors. ^c ^Single study reporting male cases: 1/22

Reference	Country	Type of study	Number of patients	Age (years)	NEN type	Breast metastases as initial manifestation	Laterality	Primary site	Other metastases	Carcinoid symptoms
Papalampros et al., 2009 [14]	Greece	CR	1	52	NET	Yes	Left	Ileum	Liver	No
Glazebrook et al., 2011 [16]	USA	CS	10	56^b^	NET	Yes (1) No (4) NR (5)	Left (1) Right (2) Bilateral (2) NR (5)	Lung (1) Colon (1) Small bowel (8)	NR	Yes (5) No (5)
Hasteh et al., 2007 [20]	USA	CR	1	61	NET	No	Right	Kidney	NR	NR
Mosunjac et al., 2004 [21]	USA	CR	2	60	NET	Yes	Bilateral	Ileum	Liver and ovary	No
				57	NET	No	Bilateral	Jejunum	No	Yes
Upalakalin et al., 2006 [22]^a^	NA	NaR	15	54^b^	NR	No	Left (6) Right (6) Bilateral (2) NR (1)	Ileum (10) Duodenum (1) Pancreas (1) Lung (3)	NR	Yes (4) No (11)
			9	56^b^	NR	Yes	Left (5) Right (3) Bilateral (1)	Ileum (6) Appendix (1) Ovary (1) Unknown (1)	NR	Yes (6) No (3)
Gupta et al., 2006 [23]	USA	CR	1	52	NET	No	Left	Ileum	Liver and ovary	No
Perry et al., 2011 [24]	USA	CS	18	55^b^	NET (17) NEC (1)	Yes (2) No (16)	Left (5) Right (12) Bilateral (1)	Small bowel (9) Appendix (1) Lung (5) Stomach (1) Unknown (2)	NR	Yes (10) No (8)
Lee et al., 2017 [25]	USA	CR	2	68	NET	No	Left	Small bowel	Liver	NR
				62	NEC	No	Bilateral	Unknown	Liver	Yes
Mohanty et al., 2016 [17]^c^	USA	CrS	22	60^b^	NET (15) NEC (7)	Yes (7) No (15)	Left (9) Right (8) Bilateral (5)	Gastrointestinal (8) Lung (11) Cervix (1) Endometrium (1) Ovary (1)	NR	NR
Hwang et al., 2008 [18]	USA	CR	1	75	NET	Yes	Bilateral	Gastrointestinal	Liver, Peritoneum and lung	No
Adams et al., 2009 [26]	England	CR	1	62	NET	No	Right	Ovary	Liver	No
La Rosa et al., 2015 [27]	Italy	CR	1	50	NET	Yes	Left	Ileum	Liver	No
Shahrokni et al., 2009 [28]	USA	CR	1	64	NET	Yes	Left	Small bowel	Liver	Yes
Chodoff, 1965 [29]	USA	CR	1	72	NET	No	Right	Ileum	No	No
Bohman et al., 1982 [30]	USA	CS	1	64	NET	NR	NR	Ileum	NR	NR
Wozniak et al., 1998 [31]	USA	CR	1	47	NET	Yes	NR	Lung	No	No
Choi et al., 2011 [15]	USA	CR	1	62	NET	No	Left	Lung	Liver and CNS	No
Geyer et al., 2010 [32]	USA	CR	1	52	NET	Yes	Left	Ileum	Liver, Peritoneum and bone	No
Crona et al., 2013 [19]	Sweden	CS	20	49	NET (11) NR (9)	Yes (3) No (1) NR (16)	NR	Small bowel (11) Lung (8) Thymus (1)	Liver (12) Ovary (2) CNS (1) Skin (2) Trachea (1) Bone (1)	Yes (5) No (13) NR (2)
Strosberg et al., 2007 [33]	USA	CS	3	NR	NET	NR	NR	NR	Ovary, peritoneum and liver	NR
				NR	NET	NR	NR	NR	Ovary and Peritoneum	NR
				NR	NET	NR	NR	NR	Ovary and skin	NR
Policeni et al., 2016 [34]	USA	CR	1	66	NET	Yes	Left	Ileum	Liver	No
Amin and Kong, 2011 [35]	USA	CR	1	69	NET	Yes	Left	Unknown	Liver	Yes
O'Donnell et al., 2009 [36]	Ireland	CS	1	52	NET	Yes	Right	Ileum	NR	No

In the same way, many features of patients affected by breast metastases from NENs are not quite different from those of patients affected by NENs in general. For example, the mean age at NET diagnosis has been reported to be 58-65 years [1,9]. Likewise, it has been reported that most NETs are non-functioning [37,38], i.e., they do not produce hormone-related symptoms. On the contrary, many of these tumors are incidentally found or manifest with symptoms related to local organ damage [39]. For gastrointestinal (GI) NETs, the most reported symptoms include abdominal pain, bowel obstruction, and diarrhea. Carcinoid heart disease, flushing, and GI bleeding are far less common [39].

According to our review, most cases had their origin in the digestive tract (63%) or the lung (27%) (Table 2). Small bowel was the most frequent site of origin among digestive tract-derived NEN, whereas colonic origin seems to be infrequent, with only one reported case [17]. Gupta et al. made similar observations in their review in which they also found that the small bowel, specifically the ileum, was the most frequent origin [23].

**Table 2:** NEN primary site frequency Built with data from Table 1 a Primary site was reported in 113 out of 116 cases. b Specific site was reported in 62 out of 71 cases of primary gastrointestinal NEN. c Specific segment of small bowel was reported in 27 cases: 25 ileum, 1 jejunum, and 1 duodenum. NEN: neuroendocrine neoplasm

Primary tumor	n^a^	%
Gastrointestinal^b^	71/113	63%
Small bowel^c^	57/62	92%
Colon	1/62	2%
Pancreas	1/62	2%
Appendix	2/62	3%
Stomach	1/62	2%
Lung	30/113	27%
Ovary	3/113	3%
Kidney	1/113	1%
Cervix	1/113	1%
Endometrium	1/113	1%
Thymus	1/113	1%
Unknown primary	5/113	4%

Breast metastases from NEN are diagnosed through histologic findings. However, there are many morphologic features that overlap among these tumors and breast carcinomas (particularly neuroendocrine differentiated) that could lead to a wrong diagnosis [14,21,24,25]. Some of these NENs may go unnoticed and may even be treated as breast carcinomas [27,28]. In fact, many of the reported cases (Table 1) needed a pathology review to change diagnosis to NEN. Furthermore, Carreras et al. retrospectively evaluated 4210 68Ga-PET/CT to determine the frequency of metastases [13]. They found 21 (0.5%) patients with breast metastases, which could place breast metastases at the fifth place in frequency, behind liver, lymph node, bone and heart metastases. Thus, it is likely that the real number of breast metastases of NENs has been historically underestimated [18,19]. In this scenario immunohistochemistry is of great value: synaptophysin, chromogranin, NSE (neuron-specific enolase), PC3, CDX-2, serotonin, substance P and PGM tend to be positive in NEN; whereas estrogen receptors, cytokeratin 7, and GATA3 tend to be negative [17,23,27,35].

Imaging allowed us to suspect breast metastases. Most guidelines advocate the use of contrast enhanced CT or MRI of abdomen, pelvis and chest to rule out metastasis in NENs [2,40]. Of course, there are no pathognomonic features with them or with the traditional methods that anatomically evaluate breast: mammography, echography, and MRI [34,41-43]. Also, the few reported cases of breast NENs do not allow to generalize imaging findings [44]. Functional images, based on the presence of somatostatin receptors, are highly relevant in this scenario; specially those based on 68Ga-dotatate as radio marker, given their greater sensitivity [45]. In the report by Glazebrook et al., Octreoscan, a less sensitive functional method, was positive in just four out of five cases of biopsy-proven NEN breast metastases [16].

Optimal treatment for breast metastases from NENs is not clear given the paucity of clinical data [18,34], so they are usually managed according to the guidelines of metatstaic NENs. The main pillar of GI NET treatment is surgical resection with curative intent, if possible, with no known role for adjuvant systemic therapy [2,4,40]. Even when curative intent is not possible, noncurative debulking surgery can also be conducted to control tumor-related symptoms or hormone secretion [3]. Furthermore, some studies have suggested that resection of primary GI NET could improve survival, even in patients whose metastases are not resected [46,47]. On the other hand, chronic medical therapy is necessary when NETs are diagnosed in advanced stages, both for symptom control and for growth suppression [6,48]. The somatostatin analogs lanreotide and octreotide have proved to be useful as initial treatment for both aims [6,49,50]. Lutetium 177 dotatate is indicated as second-line therapy for these patients [51]. 

## Conclusions

We reported a patient with an ileocecal NET metastatic to both breasts, initially suspected from 68Ga-PET/CT findings. A comprehensive (non-systematic) review of all cases of breast metastases from NENs indicates that most cases correspond to NETs and originate in the digestive tract. Also, there appears to be no preferred laterality for breast metastases. A correct diagnosis is of paramount importance for proper treatment. To this end, functional imaging, and histology (supported by immunohistochemistry) are of great value. Finally, it must be emphasized that the real incidence of these cases is probably greater than previously considered.
